# Neurological complications in oncology and their monitoring and management in clinical practice: a narrative review

**DOI:** 10.1007/s00520-024-08894-5

**Published:** 2024-09-25

**Authors:** Stefanie Fischer, Malte von Bonin, Martin Bornhäuser, Christian Beste, Tjalf Ziemssen

**Affiliations:** 1 Center of Clinical Neuroscience, Department of Neurology, University Hospital Carl Gustav Carus, Technical University of Dresden, Dresden, Germany; 2 Department of Internal Medicine I, University Hospital and Faculty of Medicine Carl Gustav Carus of TU Dresden, Dresden, Germany; 3 Cognitive Neurophysiology, Department of Child and Adolescent Psychiatry, Faculty of Medicine of the TU Dresden, Dresden, Germany

**Keywords:** Oncology, Tumor, Neurotoxicity

## Abstract

**Importance:**

New anti-tumor treatments, such as immune checkpoint inhibitors and CAR T-cell therapy, are associated with an increasing number of neurological issues linked to tumors not arising from nervous system such as neurological and neuropsychological side effects that can significantly impair quality of life in the short or long term. The science of pathomechanisms, therapeutic approaches, and preventive measures is still in its early stages, and the progress is hampered by the lack of studied connection between neurological and oncological disciplines.

**Objectives:**

This work aimed to provide an overview of the questions raised in the field of clinical neuroscience that concern the outcomes of oncological diseases and their treatment. Furthermore, we give an outline of how a collaborative approach between neurology and oncology, with the implementation of neuroscience techniques including up-to-date diagnostics and therapy, can help to improve the quality of oncological patients’ lives.

**Evidence review:**

The covered areas of investigation in the evaluated articles primarily encompassed the review of known neurological complications of oncological diseases caused by neurotoxic mechanisms of performed therapies or those linked to concurrent pathological conditions. Similarly, the methods of their diagnostics were assessed.

**Findings:**

Our literature review of 65 articles, including clinical trials, cohort studies, reviews, and theoretically based in vitro studies published between 1998 and 2023, outlines the broad spectrum of neurological complications primarily associated with malignant diseases and the anti-tumor therapies employed. Notably, immune-mediated complications, whose incidence is increasing due to the expanding use of new immunotherapies, require early detection and targeted treatment to prevent severe progression. In this context, neurological complications mediated by immune checkpoint inhibitors are often associated with significant impairments and high mortality, necessitating specialist consultation for early detection and differentiation from other phenotypically similar syndromes. Current data on the pathophysiology of these neurological complications are not reliable due to the limited number of studies. Moreover, there is a lack of evidence regarding the appropriate oncological approach in the event of therapy-related complications. Initial study results suggest that the establishment of interdisciplinary treatment interfaces for the management of oncology patients could improve the safety of these therapies and enhance the patients’ quality of life.

**Conclusions and relevance:**

The accumulated knowledge on neurotoxicity caused by oncological diseases shows that the challenges in diagnosing and managing this condition are expanding in tandem with the growing array of therapies being employed. Therefore, it requires interdisciplinary approach with the introduction of new facilities enabling more personalized patient care.

## Introduction

The definition of modern neuro-oncology has evolved with respect to an increasing number of partly life-threatening neurological complications arising as a result of new anti-tumor therapies in patients with malignancies unrelated to the nervous system.

The gains in life expectancy from new therapy regimens urge the improvement of the quality of life for this category. This requires a better understanding of the underlying mechanisms of neurotoxicity and the use of evidence-based diagnostic and therapeutic measures. Improved cooperation between neuroscience and oncology in research, diagnosis, and treatment is therefore essential.

**Fig. 1:**
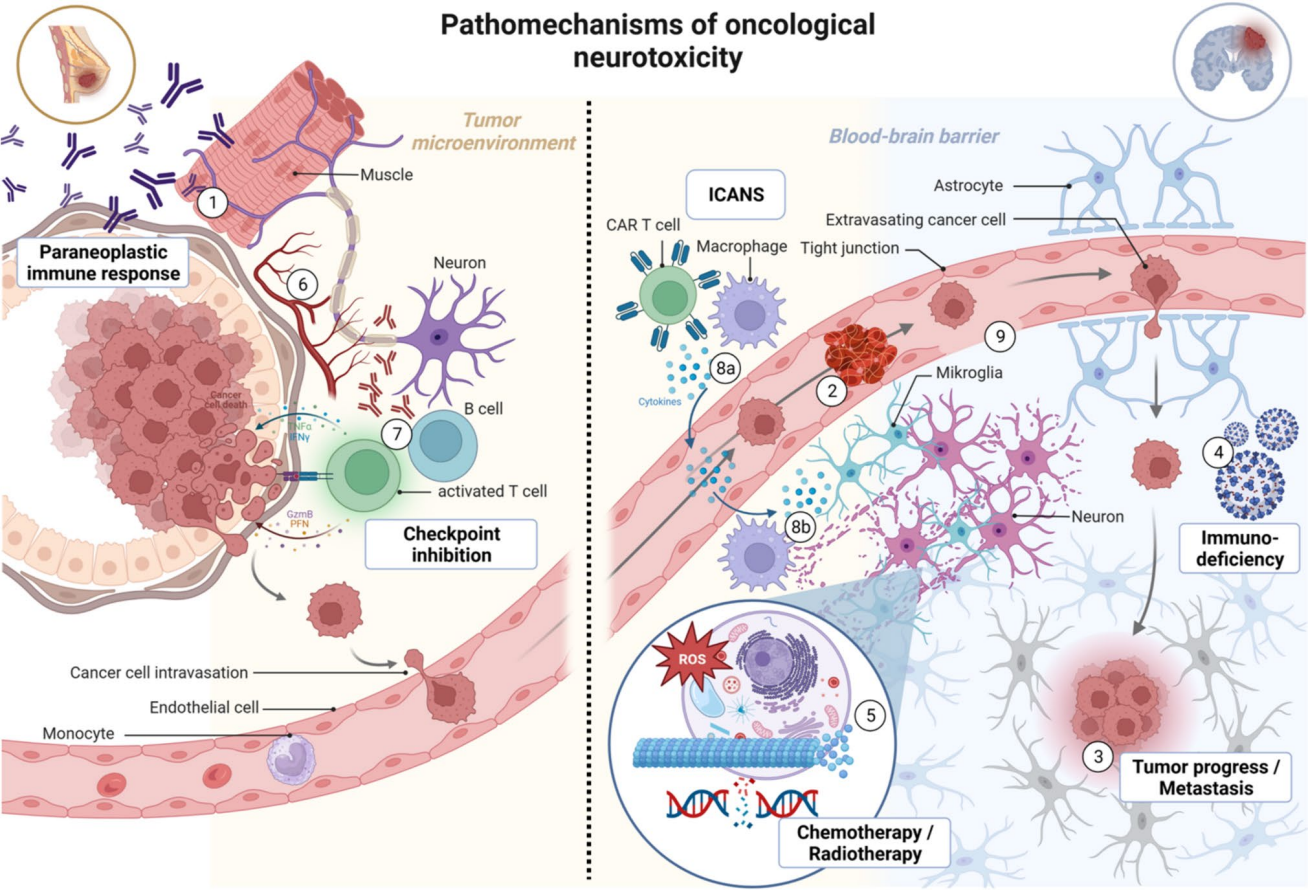
Pathomechanisms of neurotoxicity in malignoms using the example of breast cancer: (1) cellular and humoral-mediated neuroinflammation in the context of paraneoplastic syndromes; (2) ischemic stroke due to paraneoplastic thrombophilia; (3) CNS metastasis; (4) opportunistic CNS infection; (5) direct cytotoxic effects by chemotherapeutics and radiation (e.g., DNA damage, disturbance of axonal transport and other cell organelle functions); (6) ischemia of nerve structures, due to compression by the tumor, endothelial damage by toxic metabolites, or ionizing radiation or angiogenesis inhibition due to VEGF binding; (7) disturbance of immune tolerance through blockade of CTLA4, PD-1, and PD-L1; (8a) cytokine-mediated damage due to non-specific activation of macrophages and monocytes followed by disturbance of the blood–brain barrier; (8b) cytokine-mediated excitotoxicity through microglia, neuroinflammation due to local CNS internal cytokine production; (9) endothelial dysfunction and disruption of blood brain barrier by chemotherapy, radiation, or CAR T cell therapy

In the following section, we provide an overview of the spectrum of neurological complications in oncology and their pathomechanisms (Fig. 1). The second part of this paper gives an outline of how a new interface between neurology and oncological disciplines can improve acute and long-term care of oncological patients.

## Clinical spectrum of neurotoxic complications

### Neuronal damage caused by the underlying pathologic processes in oncological diseases

Apart from direct mechanical alteration caused by the presence of malignant tumors at primary site, metastases, and meningeosis, paraneoplastic syndromes can be an indirect cause of neurological complications in oncological patients. Paraneoplastic syndromes affecting the nervous system, occurring in approximately 1/300 of all cancer patients and can manifest in the form of encephalomyelitis, limbic encephalitis, subacute cerebellar degeneration, opsoclonus-myoclonus syndrome, sensory neuronopathy, chronic gastrointestinal pseudo-obstruction, Lambert-Eaton syndrome, as well as dermatomyositis [1, 2]. Additionally, paraneoplastic thrombophilia can result in severe neurological deficits due to ischemic strokes, and tumor-associated amyloidosis can lead to the formation of pathological protein aggregates in and around nerve structures. Another example of indirect complications of malignant diseases are opportunistic central nervous system infections (PML, toxoplasmosis, cryptococcosis, etc.), which can result from tumor- and/or therapy-associated immunosuppression.

## Therapy-associated neurological complications

### Chemotherapy/cytostatics

The history of cytostatics begins with the chemical warfare agent sulfur mustard, after physicians discovered its antiproliferative effects during World War I [3]. The original use as a chemical warfare agent already suggests a significant potential for side effects of this drug category.

Peripheral neuropathy developed due to cytostatic drugs is the most common neurological complication of anti-tumor therapy, found in 30–70% of oncological patients and significantly contributing to their morbidity and impaired quality of life [4, 5]. Platinum-containing drugs, taxanes, vinca alkaloids, and proteasome inhibitors are among the cytostatic drugs which have proved to activate the mechanisms of peripheral neuropathy [6].

The severity of neuropathy depends on the various factors, such as the mechanism of action of the cytostatic agent used, the cumulative dose, the duration of use, pre-existing damage to nervous system, and the vulnerability of respective peripheral fiber entities to the chemotherapeutic agent (dorsal root ganglia, distal nerve fibers, motor or autonomic fibers). The most common manifestations of neuropathy include par-, dysaesthesias, allodynia, and hyperalgesia of hands and feet. The exposure to vinca alkaloids, such as vincristine, can lead to primary impairment of motor and autonomic fibers in turn [7]. In addition to significantly impaired quality of life, autonomic dysfunction can also have life-threatening consequences.

Cytotoxic agents can also affect central nervous system in multiple ways. Aseptic meningitis or acute encephalopathy have been reported to appear in 10–60% of cases and can be transient [6]. Symptoms can include headache, signs of meningeal irritation, confusion, disorientation, hallucinations, fatigue, or a risk of seizures, where the mechanisms of direct toxicity, folate depletion, and dysfunction of blood–brain barrier are thought to play a causal role [6].

Another pathologic condition is a vascular pathology affecting brain-supplying vessels and presenting as ischemic stroke, intracranial hemorrhage, or venous thrombosis [6].

Cerebellum, hippocampus, and white matter of brain are particularly vulnerable to cytotoxic agents. Then, 10–20% of cytostatic-treated patients develop cerebellar neurotoxicity, of which a quarter subsequently retain permanent cerebellar deficits [6].

“Chemobrain” or “chemofog” is a term frequently used by patients to describe the cognitive disturbances, such as impaired concentration, reduced processing speed, memory impairment, and deficits in executive function following chemotherapy. And 40–80% of patients treated with chemotherapy may experience this cognitive impairment [8]. Pathophysiologically, the cognitive impairment may be related to the loss of neural precursor cells, oxidative stress, inflammation, and neurovascular injuries [6].

Damage to white matter tracts often leads to personality changes, motor and coordination impairments, and urinary incontinence. In some cases, patients may develop leukoencephalopathy and progress to moderate-to-severe dementia [9]. The highest incidence of delayed leukoencephalopathy occurs in those treated with high-dose or intrathecal methotrexate (10–40%), particularly causing more problems in CNS lymphoma or leukemia [10, 11].

### Radiation

Neurological deficits linked to radiation often affect patients with primary CNS tumors or brain metastases, as CNS structures are located directly in the radiation field.

Regarding radiation-induced neurological damage, a subdivision into acute and long-term complications can be made. Acute complications are usually transient and manifest as radiation-induced encephalopathy with mental status changes, fatigue, or worsening of pre-existing neurological symptoms [6]. Underlying pathophysiological mechanisms are still insufficiently investigated; however, disruption of blood–brain barrier, development of brain edema, inflammatory changes, and ischemia due to radiation-induced vascular toxicity are suggested [12].

The most challenging complications, however, usually manifest in a chronic-progressive manner, where cognitive deterioration, sometimes with the development of dementia syndromes, is present in up to 80% of cases [8]. The main causes are considered to be radiation-induced cytotoxic effects with tissue necrosis, leukoencephalopathy, and subsequent brain volume reduction, damage to the blood–brain barrier with chronic neuroinflammation, and injury to brain-supplying vessels [13]. Cerebrovascular complications are also among the delayed side effects of radiation appearing in about 15% of all cancer patients [6]. In patients who are irradiated due to a head or neck tumor, symptomatic stenosis of the internal carotid artery occurs in up to 50% of cases [14]. The development of cerebral microhemorrhages and vascular malformations due to radiation is also possible and should be considered, especially before starting an anticoagulation [15].

### Angiogenesis inhibitors

Angiogenesis inhibitors like bevacizumab, sorafenib, and sunitinib therapies can disrupt the integration of (cerebral) blood vessels and thus cause ischemia or intracranial bleeding. Furthermore, symptoms can occur as a result of endothelial dysfunction (e.g., PRES) [6].

### Immunotherapy

#### Immune checkpoint inhibitors

Immune checkpoint inhibitors (ICI) are an oncological treatment approach that enhances the body's antitumor immunity by modifying regulatory proteins (checkpoints). Despite their high efficacy in treating various cancers, the intense immunological activation they induce can often disrupt immunological self-tolerance, leading to the development of immune-related adverse events (irAEs), which can affect any organ system. Although neurological irAEs are relatively rare, with an incidence of 1–5%, they, along with cardiac irAEs, represent complications with the highest mortality rates (8–14%) [16]. Furthermore, the severity of neurological irAEs often necessitates the permanent discontinuation of immune checkpoint inhibitors, followed by intensive immunosuppression, which may carry an unknown risk for tumor progression. However, there are currently few studies that systematically document the clinical outcomes of these patients, making evidence-based decisions on long-term treatment strategies challenging [17]. Additionally, differentiating between primary paraneoplastic syndromes and irAE-associated paraneoplastic syndromes is clinically challenging [16, 18]. While there are some general distinguishing criteria, it is crucial to accurately identify the etiology due to the differing pathophysiologies and the subsequent need for distinct therapeutic approaches [16]. This should always be done with neurological expertise.

Initial studies have shown that establishing interdisciplinary care for patients treated with ICI can reduce hospitalization rates and mortality, emphasizing the importance of targeted, expert diagnostics and the early initiation of appropriate therapies [19].

#### CAR T-cell therapy

CAR T-cell therapy is widely recognized as a highly effective treatment for various hematological malignancies. In addition to cytokine release syndrome, neurotoxicity in the form of ICANS affects 60–80% of patients undergoing CAR T-cell therapy and manifests in the various ways, from mild encephalopathy to potentially life-threatening brain edema with coma [20–22]. The subjacent pathomechanisms are not fully understood yet, but the cytokine-induced disruption of blood–brain barrier and a direct neurotoxic effect on neurons and glial cells are discussed to play a leading role [23]. The symptoms usually occur 3–10 days after CAR T-cell infusion and typically resolve within 7–10 days with appropriate therapy [6, 23]. The long-term neurological prognosis appears to be good, but reliable data on outcomes are lacking.

## Reality of practical care and scientific understanding of oncological neurotoxicity

When it comes to oncological neurotoxicity, there is a significant uncertainty in dealing with neurological symptoms and the further oncological therapy procedures [24–27], which often leads to hospitalization and leaves patients with significantly impaired quality of life [13, 28–31].

In the context of studies, neurological toxicity is currently recorded as adverse events (AE) according to CTCAE classification or in the case of ICANS as ICE score [32–37]. However, the CTCAE classification is not eligible to consider functional outcomes. The inadequate collection of neurological outcomes results in a lack of clinical studies with convincing outcomes on neurotoxic complications caused by oncological therapy or on possible preventive measures.

As today’s oncological therapy repertoire offers diverse therapeutic approaches, their neurological outcomes should be systematically investigated, and the questions regarding the most favorable point of time for their use with respect to neurotoxicity should be examined (e.g., Is the early use of CAR T-cell therapy safer in the absence of pre-existing, chemotherapy-associated damage?).

## Opportunities in modern neuroscience for oncological care

**Fig. 2:**
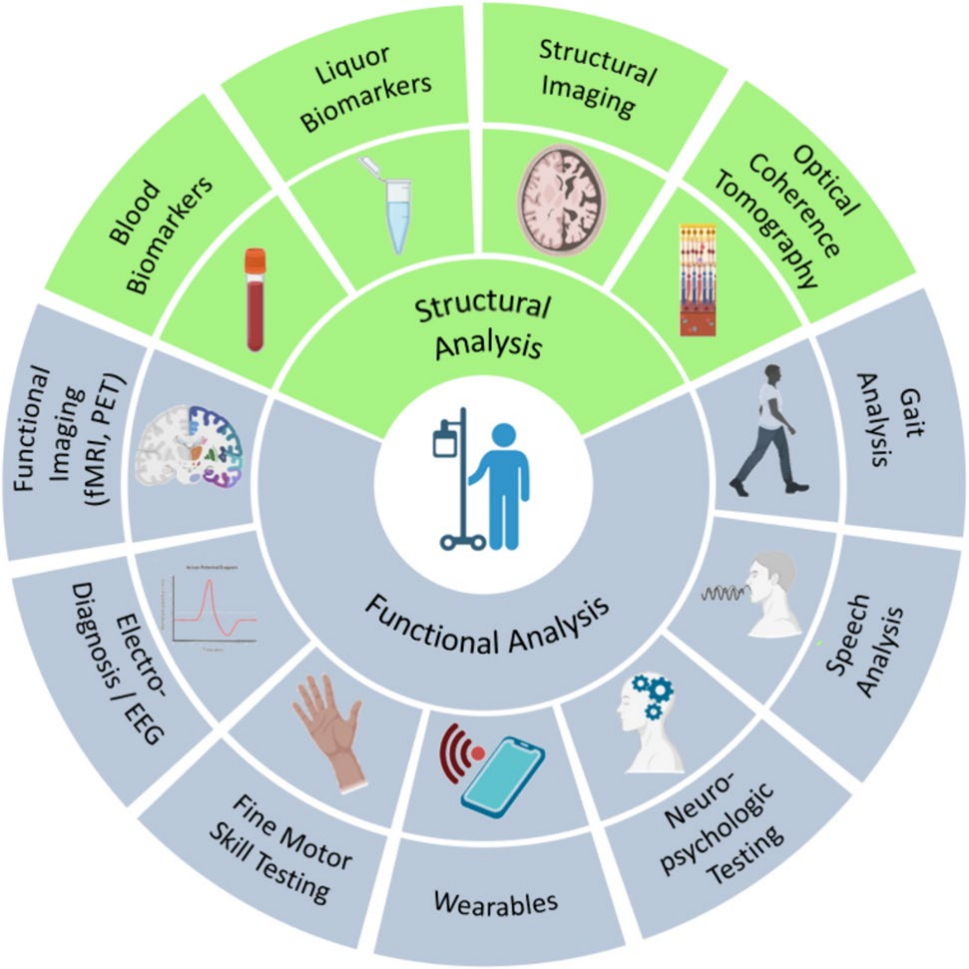
Assessment tools of neurological outcomes

While the assessment of neurological functional status was previously limited to the relatively subjective clinical examination, today there are numerous supplementary diagnostic tools available, which should be presented in the following. Finally, Fig. 2 provides a schematic overview of the various analysis parameters. These tools not only allow for obtaining quantifiable and easily comparable findings but also enable the assessment of function-based outcomes, which measure how a patient performs specific activities of daily living [38]. With the advancement of neurological diagnostics, the possibility of personalized medicine emerges, where patients can be stratified according to broader characteristics. The assessment of functional neurological outcomes also provides an opportunity to conduct studies with the valid outcomes regarding neurotoxicity independently from the CTCAE system.

### Cerebrospinal fluid (CSF) analysis

#### CSF-based investigation of ICANS

While the clinical features of ICANS are easily recognizable, its pathology remains poorly understood and is based on findings from animal models. Recent research in mice and non-human primates has suggested that endothelial cell activation and disruption of the blood brain barrier result in direct neuronal cell injury, in addition to the involvement of various pro-inflammatory cytokines [39]. However, the role of CAR T-cells themselves in the development of neurotoxicity remains unclear. They are found in both established cases of ICANS and in cases with no apparent symptoms in the CSF. While some researchers consider them active players in the development of neurotoxicity, others attribute them to a bystander role and regard other mechanisms as causative, such as microglial activation with excitotoxicity [3]. ICANS did not manifest in early mouse models but emerged during clinical trials. Therefore, it becomes even more important to understand the precise pathomechanisms in the human central nervous system and to verify the findings obtained through animal models. For these inquiries, a standardized determination of intrathecal cytokine repertoires; neurotransmitters; neurodestruction markers; parameters of leukocyte infiltration like CXCL13, CXCL10, MMP-9; as well as a differentiated cerebrospinal fluid cell analysis under ICANS would be valuable [40].

The exact understanding of the pathomechanisms of ICANS is fundamental to developing tailored CAR T-cell products and neuroprotective agents, as well as directed ICANS therapies. Moreover, the precise clarification of pathomechanisms and processes in the cerebrospinal fluid compartment during peripheral or intrathecal CAR T-cell administration is likely to play a significant role in the treatment of oncological diseases with CNS involvement.

#### CSF-based investigation of “chemobrain”

Due to its profound consequences and irreversibility, cognitive impairments known as chemobrain or chemofog have significant patient-specific and socio-medical relevance. However, there are only few insights into the exact mechanisms of its development. Potential mechanisms of chemotherapy-induced damage discussed so far include the impairment of neuronal precursor cells, oxidative changes, inflammation, and neurovascular injuries [6–8, 18]. A precise clarification of the pathomechanisms could be achieved through CSF-based standardized measurements, including “classic” neurodestruction markers, as well as tissue damage-related inflammation parameters like osteopontin and CH13L1 [40], and regenerative parameters providing information about the neurons’ regeneration capacity [41].

Other interesting scientific questions could include whether the observed cognitive decline after radio- or chemotherapy is additionally due to secondary neurodegeneration related to tau, amyloid, or synuclein pathology, which should be treated accordingly, or if it is solely a result of direct toxic effects of chemotherapy.

#### Serum-based biomarkers

Advancements in ultra-sensitive immunoassay technologies have enabled the detection of many neurological biomarkers in serum. Particularly, neuronal destruction markers, such as Neurofilament light chain (NfL), S100B, GFAP, MCP-1, NSE, and BDNF [42], can now be reliably quantified in the blood. These biomarkers offer a good monitoring option for patients who do not undergo lumbar puncture.

NfL has now become well-established as a cross-disease blood-based biomarker that is highly sensitive to neuronal damage. It has been associated with disease severity and prognosis in multiple neurological diseases and general neurodegeneration [43–45]. Even though changes in NfL levels in biofluids do not reflect any specific mechanism of damage, this biomarker may have diagnostic value in terms of monitoring of axonal loss [45], which may be important for detecting neurotoxicity in oncological patients as well. For example, recent studies have found that serum NfL levels are elevated in most patients with confirmed ICI-induced encephalitis, which could be helpful in ruling out differential diagnoses given the often heterogeneous clinical presentation [46]. NfL measurement could represent an initial step for quickly identifying patients who may require secondary procedures (such as brain MRI or lumbar puncture). Moreover, current results suggest that NfL levels could also aid in predicting the response to irAE treatment and may be useful in identifying patients who might need immunosuppressants in addition to steroids [46].

Alongside NfL, a recent study highlights the diagnostic importance of neural antibodies, particularly paraneoplastic antibodies in focal ICI-encephalitis and anti-GFAP antibodies in meningoencephalitis. When present, paraneoplastic antibodies were independently associated with a lack of response to irAE treatment, suggesting that they can be used not only for diagnosis but also for prognosis, in conjunction with NfL levels [16]. Importantly, neural antibodies in patients with post-immune checkpoint inhibitor paraneoplastic neurological syndromes are sometimes detected before treatment, indicating that these antibodies might help predict the development of neurological adverse events [16]. Further research in this field is urgently needed.

### Imaging

Cerebral imaging has already proved its significance in oncology for the detection of tumor progression or infection-related complications. Recent advances in quantifying software have facilitated highly precise assessment and intra- or inter-individual comparison of various changes, including ischemic or inflammatory alterations [47–49]. The application of quantitative cerebral imaging in neurology can be beneficial in the oncological sector for the precise quantification of brain volume reduction and leukoencephalopathy. This approach can be useful for individual disease monitoring and standardized examination of medication side effects in a research setting, such as comparing leukoencephalopathy development under different chemotherapy regimens or radiation schemes.

### Gait analysis

Numerous neurological conditions are reflected in early changes in gait patterns. For example, studies have shown that a decline in memory function correlates with a reduction in walking speed [50]. Additionally, progression in multiple sclerosis can be sensitively and early detected using gait analysis [51, 52].

Gait disturbances in oncological patients can be diverse and multifactorial, manifesting with spinal ataxia and distal weakness due to chemotherapy-induced polyneuropathy, cerebellar ataxia due to paraneoplastic syndromes or CAR T-cell neurotoxicity, and neurodegeneration-related deterioration of gait due to chemotherapy- or radiation-induced leukoencephalopathy. Therefore, objectively assessing these disturbances as functional outcomes can be beneficial for individual patient care (e.g., in terms of objective clinical monitoring, appropriate assistive device selection, and objective assessment of treatment response).

### Fine motor skills testing

Impairments in fine motor skills are often observed in oncological patients, resulting from chemotherapy-induced polyneuropathy, CAR T-cell- or immune checkpoint inhibitor-related neurotoxicity, or paraneoplastic syndromes. From a neurological perspective, standardized assessment can be helpful for monitoring the progression of neurotoxic complications, quantitatively examining scientific inquiries, as well as assessing the level of disability and the need for social-medical support. Moreover, the response to therapeutic interventions can be reliably evaluated through this method.

### Optical coherence tomography (OCT)

Optical coherence tomography (OCT), a non-contact, non-invasive imaging modality that relies on measuring echo time and scattering of infra-red light, is commonly utilized in neurology for evaluating axonal loss and damage related to optic neuritis [39, 40]. Likewise, in multiple sclerosis, certain researchers have also noted substantial decrease in retinal nerve fiber layer thickness (RNFL), even in clinically unaffected eyes [53], and have shown that the latter measured by OCT correlates with the extent of neural degeneration in brain Therefore, RNFL thickness measured by OCT is already used as a surrogate parameter to monitor axonal loss and neural degeneration. As the assessment is simple, non-invasive, standardized, and generates quantifiable results, it can be a valuable addition in the monitoring of oncological patients who are likely to develop axonal loss or are at risk of neurodegeneration, for example, due to high-dose chemotherapy.

### Electrophysiology

In the setting of oncological neurotoxicity, EEG is already used in the acute diagnosis and management of ICANS in CAR T-cell therapy patients. Valuable scientific inquiries arising from regular EEG application in CAR T-cell patients could include identifying high-risk patients for ICANS development through pre-treatment EEG screening and assessing potential benefits of seizure-suppressing medication.

Electroneurography and electromyography can play a role in objectively assessing the extent and pattern of damage in neuro-muscular pathologies. In the oncological context, they already play an important role in differentiating immune-mediated phenomena in terms of neuro-muscular transmission disorders (e.g., immunotherapy-related Myasthenia Gravis, Lambert-Eaton syndrome), neurogenic injury (e.g., immune checkpoint inhibitor-mediated GBS or CIDP, paraneoplastic neuropathies), or muscular damage (immunotherapy-mediated myositis). For scientific investigation of potential prevention measures for chemotherapy-induced PNP, repeatedly and standardized conducted neurography would be essential.

Tremor analysis is also a well-established electrophysiological diagnostic tool in neurology. It allows for the identification of peripheral or central tremor generators and provides an objective assessment of (neurotoxicity-induced) tremors over time.

### Speech analysis

Many neurological disorders have noticeable or subtle effects on language and articulation [54–56]. Changes in diction and speech can now be objectively measured (e.g., through app-based tools) and can provide early indications of disturbances in neural processes. Furthermore, they can be used for the evaluation of aphasic and dysarthric symptoms over time. Potential applications include objective assessment and early detection of a scanning speech pattern concerning paraneoplastic cerebellar syndromes or vocal tremors resulting from ICANS.

### Neuropsychological testing

Neuropsychological and cognitive deficits are often challenging for affected individuals to grasp verbally, yet they come with a significant burden, sometimes manifesting in nonspecific ways and are usually perceived by the patients’ environment only when they have advanced. An objective and early assessment of neuropsychological impairments can be achieved through standardized neuropsychological test batteries, where the worse performance in oncological patients can be linked to volume reduction in grey matter and decreased neuroplasticity [74,75].

The value of regular neuropsychological testing for oncological patients during the course of their illness and treatments is evident. By assessing the complaints associated with “chemobrain,” specific therapeutic measures can be elaborated (e.g., occupational therapy, inpatient rehabilitation, antidepressant treatment), and the need for social-medical interventions, such as applying for disability benefits or a disabled status, can be fulfilled. Additionally, neuropsychological testing can play a key role in essential scientific inquiries, such as the prevention of cognitive side effects from oncological therapies.

### Wearables/digital twin

Digital phenotyping is making its way into the field of neurology, with the goal of identifying previously undiscovered patterns, predicting functional outcomes, and simplifying neurological treatment planning [57, 58]. They enable continuous, longitudinal health monitoring outside of the traditional clinical setting, also in CAR-T cell therapy [78]. In addition to patient monitoring, wearables also facilitate the development of algorithms for automated health event prediction, prevention, and intervention as well as potentiality could expand the scope of neurological diagnostics.The development of digital twins holds significant potential for advancing personalized management of neurological adverse effects resulting from oncological therapies [59]. Through artificial intelligence-driven analyses of various disease parameters—including clinical and para-clinical outcomes, multi-omics data, biomarkers, patient-related information, lifestyle factors, and medical interventions—a digital twin mirroring the patient's unique characteristics can be constructed. This digital counterpart would enable healthcare professionals to effectively manage vast amounts of patient data. By integrating information from multiple sources in a standardized fashion, healthcare providers can deliver more tailored and effective care. This approach facilitates individualized treatment plans, enhances communication between physicians and patients, and promotes shared decision-making, ultimately improving patient outcomes.

## Integrated neuro-oncological inpatient care

For meeting the aims of complex approach to diagnostics and treatment of neurotoxicity in oncological patients, neurological and neuropsychological assessments have to be initiated early in the course of disease, at least prior to the beginning of an anti-tumor therapy.

**Fig. 3:**
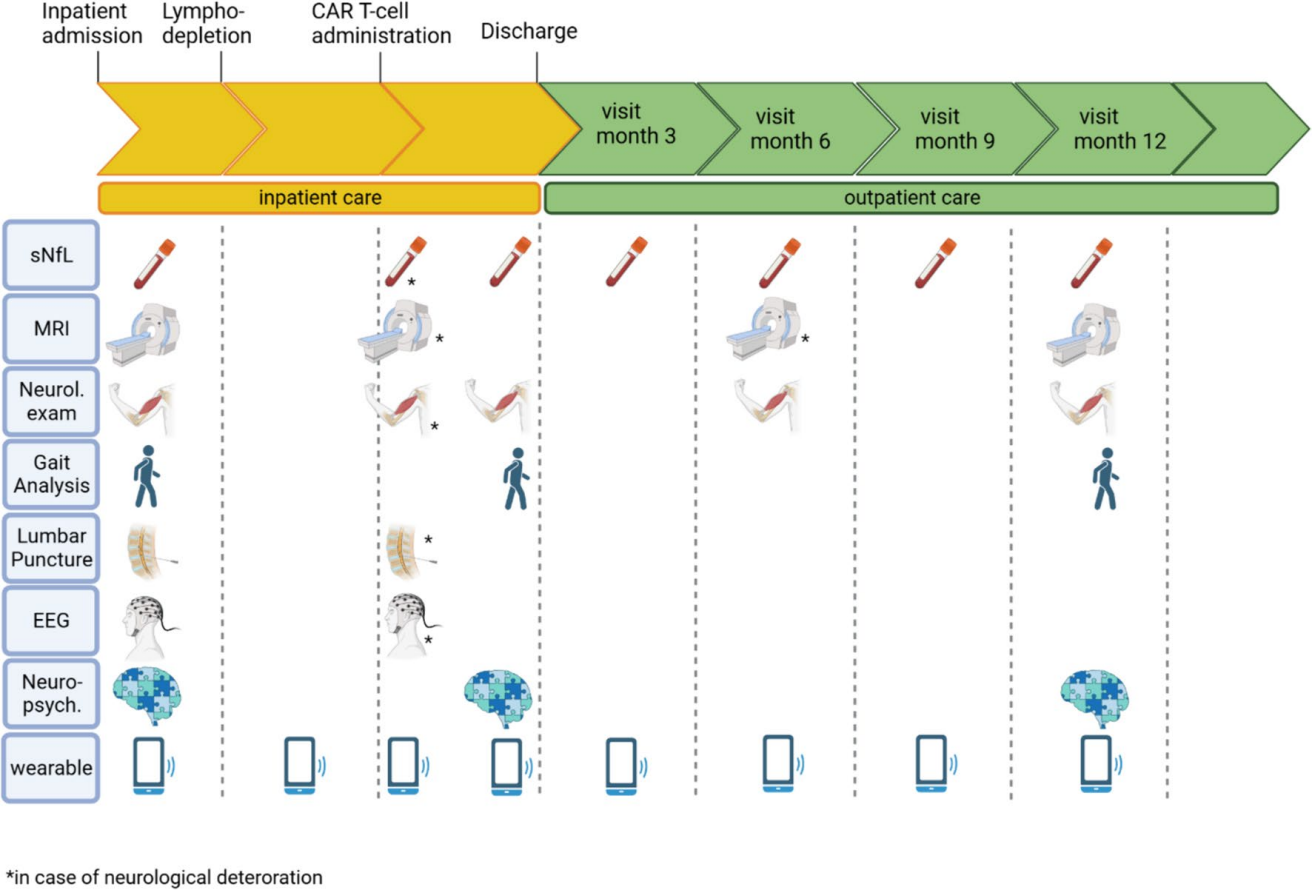
Example of a patient pathway for neurological monitoring during CAR T-cell therapy. Upon admission to hospital, a patient undergoes comprehensive baseline assessment, which serves as a comparative reference in case of sudden neurological decline and for regular follow-up assessments

Based on the risk profile, additional diagnostic tests such as EEG, electrophysiological examinations, or cerebral/spinal imaging may be required to classify pathological findings during or after the treatment cycle or to take specific preventive measures before the initiation of therapy, like an adjustment of dosage of anti-seizure medication. In case of acute neurological complications, close interdisciplinary cooperation should be guaranteed, enabling to conduct targeted diagnostics. At the end of the hospital stay, a neurological final examination with recommendations for further outpatient treatment, e.g., regarding physiotherapy or medication adjustment, can contribute to advance recovery of oncological patients, resulting in an improvement of their quality of lives. Figure 3, which can be find at the end of this section, illustrates an exemplary patient pathway for neurological monitoring during CAR T-cell therapy, which serves both patient care and scientific inquiries.

## Integrated neuro-oncological outpatient care

Rehabilitation treatment or the prescription of physiotherapy, ergotherapy, or speech therapy seems to be trivial measures, but are seldomly realized during the outpatient treatment course, often due to a lacking consideration of the ambulant physician.

The lack of holistic care after anti-tumor therapy can be compensated by the implementation of an integrated neuro-oncological outpatient department. An interdisciplinary outpatient clinic with the focus on oncological and neurological issues should assess the oncologic therapy response but also be in charge of standardized neurological diagnostics. The regular application of standardized examinations like neuropsychological testing, quantifying imaging, or neurological biomarkers as mentioned above are valuable to monitor for neurotoxic complications [60].

Based on neurological follow-up examinations, targeted symptomatic therapies can be initiated, such as mental training measures, the treatment of neuropathic pain, or the prescription of specific physiotherapy. Furthermore, neurological status can be considered in further planning of oncological therapy in case of tumor progress. Prospectively, digital phenotyping or the creation of a digital twin could also help to personalize therapy and identify potential risk factors based on specific patterns in neurological evaluations.

Modern anti-tumor therapy should include the development of an individual risk stratification in terms of neurological complications. Several risk factors regarding the deterioration of chemotherapy-induced neuropathy are already known (advanced age, pre-existing neuropathy, comorbidities like alcohol abuse or diabetes mellitus, vitamin deficiency, endocrinopathies, specific genetic polymorphisms) [61]. However, these data are not gathered systematically. Furthermore, there is a lack of reliable biomarkers for predicting the development of complications of cancer immunotherapies [16]. The results of a recently published study demonstrate that an interdisciplinary interface contributes to improved care for patients undergoing ICI therapy [19]. Regular neurological co-management of this patient group significantly reduced mortality and readmission rates due to irAEs [19]. Furthermore, this approach advanced the systematic collection of scientific data, which is a fundamental prerequisite for research in the field of neurological irAEs [19].

The range of measures and medication for the therapy and prevention of neurotoxicity is currently very limited, especially due to the lack of neurological basic research in this field. A joint care structure with standardized neurological assessments can quickly find evidence-based solutions to the various questions regarding oncological neurotoxicity. Particularly, relevant questions concerning medications and measures against oncological neurotoxicity, their influence on the response to oncological therapy and appropriate prevention measures, can be answered.

The following fictional examples intend to provide a vision of a future neuro-oncological care structure and to illustrate the diversity of intersections between neurology and oncology:

- Example 1: A patient with advanced lymphoma presents for CAR T-cell therapy. In addition to structural epilepsy after stroke, the patient has developed a length-dependent peripheral neuropathy due to several chemotherapy cycles as well as cognitive and amnestic deficits that impair daily life. In the inpatient setting, the patient undergoes a general neurological baseline examination, a neuropsychological testing, a cerebral MRI, NfL determination, and an EEG, on the basis of which the seizure-suppressing medication is administered prior to CAR T-cell therapy. Seven days after CAR T-cell therapy, the patient develops severe ICANS with recurrent epileptic seizures. MRI shows a mild ubiquitous barrier disturbance without any other changes from the initial findings. In addition, there is a significant increase in NfL. After ruling out all relevant neurological differential diagnoses, high-dose methylprednisolone therapy is initiated immediately. The seizures cease with a readjustment of the seizure-suppressing medication. Regular neurological follow-up examinations show rapid improvement of symptoms. The mild sensorimotor hemiparesis can be classified as pre-existing based on findings of baseline examination. Due to ongoing fine motor impairment and tremor symptoms, a neurological rehabilitation measure is initiated. However, the patient is readmitted 1 week after discharge with suspected biphasic ICANS. A comparison of the neurological findings cannot objectively confirm any neurological deterioration, and the neurofilament has returned to its baseline level prior to CAR T-cell therapy. After ruling out infectious or metabolic complications, the patient is diagnosed with suspected chronic fatigue syndrome, and rehabilitation measure can be continued. Three months later, the patient presents for clinical follow-up in the neuro-oncological outpatient clinic. A described deterioration in memory function can be objectively verified by neuropsychological testing. However, the testing also suggests possible causal depression. Under antidepressant medication, the patient’s memory function stabilizes over time which can be confirmed by the further neuropsychological examinations.
- Example 2: In neuro-oncology outpatient clinic, a female patient presents who has already received several cycles of methotrexate as part of her lymphoma treatment. She is participating in an interventional study on the prevention of cognitive dysfunction. The regular neuropsychological tests, quantitative cMRI examinations, and repeated neurofilament assessments have so far shown stable findings under the study medication.
- Example 3: At the same time, the further therapy procedure for a patient with bronchial carcinoma is decided based on the results of digital phenotyping. The applied digital risk stratification identifies the patient as highly susceptible for developing severe cognitive deficits under cytostatic treatment. Therefore, oncologists carefully consider possible alternative therapies.
- Example 4: A patient with urothelial carcinoma develops unilateral ptosis as an immune-related myasthenia-like syndrome under medication with avelumab. The tumor has so far responded well to the treatment. Since the myasthenic symptoms are currently mild according to neurological assessment, the therapy can be continued under close neurological monitoring, being aware that the further ICI treatment could aggravate the neurological syndrome. Furthermore, this patient requires careful screening for possible myocardial involvement that could be initially subclinical.

## Having no clean record: the role of neurons in the pathogenesis of malignancies

In the end, the recognition of another relationship between the nervous system and malignancies also demands a closer collaboration between neurology and oncology for further research and targeted therapeutic approaches. Recent studies indicate that nerve cells play a crucial role in the initiation and dissemination of tumors. Neurons and glial cells form direct communication with malignant cells within the tumor microenvironment using paracrine factors and, in certain instances, through neuron-to-cancer cell synapses-like structures [62, 63]. Distinct from direct, bona fide synaptic interactions, indirect perisynaptic contacts—reminiscent of the position an astrocyte normally assumes in a tripartite synapse—have been observed in both breast cancer brain metastases and adult glioblastoma [64, 65]. In breast cancer brain metastatic disease, glutamatergic signaling via these perisynaptic structures promotes tumor growth through NMDA receptors on the breast cancer cells [64]. Another mechanism of neuron-mediated tumor progression involves the loss of function in the p53 gene, which occurs in multiple types of cancer and leads to the reprogramming of nerve cells into adrenergic subtypes [60]. Consequently, the resulting secretion of nor- and epinephrine further stimulates tumor progression [60].

Increased understanding of nervous system-cancer interactions is beginning to elucidate therapeutic targets for various types of cancer. While these targets may vary depending on the specific tumor, exploring the neuroscience of cancer—an endeavor that requires interdisciplinary collaboration among neuroscience, developmental biology, immunology, and cancer biology—could advance effective therapies for many of the most difficult-to-treat malignancies.

## Conclusion

The close intersection of the fields of oncology and neurology, including neuropsychology, is crucial for enhancing acute and long-term care of patients with oncological neurotoxicity. The establishment of a dedicated interface that employs a standardized neurological assessment is the integral part of this process. The results gathered during multi-faceted monitoring and intensified collaboration can also advance the pressing scientific questions regarding neurotoxicity and other important neuro-oncological issues.

## Data Availability

No datasets were generated or analysed during the current study.

## Abbreviations

AE: Adverse event
BDNF: Brain derived neurotrophic factor
CAR: Chimeric antigen receptor
CH13L1: Chitinase-3-like protein 1
CIDP: Chronic inflammatory demyelinating polyneuropathy
CNS: Central nervous system
CNTF: Ciliary neurotrophic factor
CRCI: Cancer-related cognitive impairment
CRS: Cytokine release syndrome
CSF: Cerebrospinal fluid
CTCAE: Common Terminology Criteria of Adverse Events
CTLA4: Cytotoxic T-lymphocyte-associated Protein 4
CXCL10: C-X-C motif chemokine 10
CXCL13: C-X-C motif chemokine 13
DNA: Deoxyribonucleic acid
EEG: Electroencephalogram
e.g.: Exempli gratia
etc.: Et cetera
GBS: Guillain-Barre syndrome
GFAP: Glial fibrillary acidic protein
ICANS: Immune Effector Cell Associated Neurotoxicity
ICE: Immune Effector Cell-Associated Encephalopathy
ICI: Immune checkpoint inhibitors
irAE: Immune-related adverse event
MCP-1: Monocyte chemoattractant protein-1
MMP-9: Matrix metallopeptidase 9
MRI: Magnetic resonance imaging
MS: Multiple sclerosis
N-CAM: Neural cell adhesion molecule
NfL: Neurofilament light chain
NSE: Neuron specific enolase
OCT: Optical coherence tomography
PET: Positron emission tomography
PD-1: Programmed cell death protein-1
PD-L1: Programmed cell death proteinligand-1
PML: Progressive multifocal leukoencephalopathy
PNP: Peripheral polyneuropathy
PRES: Posterior reversible encephalopathy syndrome
pTAU: Phosphorylated tubulin associated unit
QA: Quinolinic acid
RNFL: Retinal nerve fiber layer
VEGF: Vascular endothelial growth factor
